# Non-Compromised Efficacy of the First Commercial Ready-to-Use Genotype 2d Porcine Circovirus Type 2 and *Mycoplasma hyopneumoniae* Vaccine

**DOI:** 10.3390/v17040554

**Published:** 2025-04-11

**Authors:** Nimród Pálmai, Nikoletta-Ágnes Széplaki, Bálint Molnár, Han Smits, Roman Krejci, István Kiss

**Affiliations:** 1 Scientific Support and Investigation Unit, Ceva-Phylaxia, 1107 Budapest, Hungary; nimrod.palmai@ceva.com; 2 Biometry Platform, Ceva-Phylaxia, 1107 Budapest, Hungary; nikoletta-agnes.szeplaki@ceva.com; 3 Bio R&D, Ceva-Phylaxia, 1107 Budapest, Hungary; balint.molnar@ceva.com; 4 Ceva Animal Health, 33500 Libourne, France; han.smits@ceva.com (H.S.); roman.krejci@ceva.com (R.K.)

**Keywords:** porcine circovirus type 2, genotype 2d, *Mycoplasma hyopneumoniae*, vaccine efficacy

## Abstract

*Mycoplasma hyopneumoniae* (*Mhyo*) and porcine circovirus type 2 (PCV2) are critical pathogens in the swine industry, both contributing significantly to the porcine respiratory disease complex (PRDC). Given their impact, it is logical to control these pathogens simultaneously. Consequently, combined vaccinations against *Mhyo* and PCV2 are gaining popularity in swine health management. We present the efficacy of the first commercial combined vaccine prepared of a genotype PCV2d strain and *Mhyo* and tested against experimental challenge infections with target pathogens in comparative trials with other commercial products. In these studies, three-week-old piglets were vaccinated according to the manufacturers’ instructions. Five weeks later, they were challenged with two *Mhyo* strains over three consecutive days or with a PCV2d strain once. Positive controls included challenged pigs without prior vaccination, while non-vaccinated/non-challenged pigs served as negative controls. The key parameters measured were lung lesion scores and seroconversion for *Mhyo*, and viraemia, rectal shedding, lymph node and lung viral content, and seroconversion for PCV2. Findings and conclusion: The results showed no compromising effects between the vaccine components and highlighted significant differences in efficacy among the various products tested. Additionally, oral fluid sampling demonstrated a strong correlation with the viraemia and fecal shedding of PCV2, underscoring the diagnostic and animal welfare benefits of this sampling method.

## 1. Introduction

Porcine respiratory disease complex (PRDC) is a multifaceted condition caused by infectious agents, management practices, and environmental, as well as host, factors [1]. Among several components, *Mycoplasma hyopneumoniae* (*Mhyo*), the causative agent of enzootic pneumonia (EP) and porcine circovirus type 2 (PCV2) infections, are of major concern [2], and typically, vaccination is practiced to control these pathogens, and, consequently, reduce economic losses, improve animal welfare conditions and herd health, and improve overall productivity [2,3].

In the field, single-dose vaccines have become more and more widespread, which presents less stress to the animals, and less labor and lower operational costs to farm staff. Furthermore, a single-dose regimen simplifies the vaccination process, increasing the likelihood of full herd coverage.

The currently available single-dose PCV2 + *Mhyo* combined ready-to-use vaccines contain either a chimera of PCV1- PCV2a (Suvaxyn Circo + MH RTU), PCV2a + b, Circomax PCV MH (Fostera Gold PCV MH), alone, or PCV2 ORF2 protein in subunit composition (Porcilis PCV M Hyo), for example [4,5,6,7].

PCV2 was first identified in the 1990s, with nearly simultaneous outbreaks in North America and Europe, and it has since achieved global distribution, largely facilitated by the movement of asymptomatic animals through the global pig trade [8].

PCV2 has a high evolutionary rate for a DNA virus, leading to the emergence of multiple genotypes [9]. These genotypes have alternated in dominance over time due to evolutionary pressures and local prevalence. Initially, the most prevalent genotype was PCV2a, which declined over time, but is still present in many regions [10]. Since the mid-2000s, PCV2b has started replacing this genotype and is still dominant in many parts of the world. The predominant, clinically most relevant PCV2 genotype currently is PCV2d; nonetheless, PCV2a and PCV2b are also circulating [7], and other genotypes such as PCV2e occur in certain regions [11].

The shifting dominance of PCV2 genotypes has necessitated updates to vaccines to ensure they remain effective against the most prevalent strains [12].

Taking into consideration the above points, a new combo vaccine was developed in a ready-to-use mixture format, which contains, for the first time on the market, and reflecting the evolution in PCV2 epidemiology, the capsid protein of a PCV2d genotype virus, together with the 2940 strain of *Mhyo*, in inactivated form.

The PCV2 capsid protein, which is the dominant immunogenic antigen, is expressed and produced using a baculovirus expression system. Besides selecting the vaccine strain, the designed manufacturing process utilizes the combination of cell lines to maximize the yield of the antigen and ensures that the two components (PCV2d and *Mhyo*) do not interfere with each other.

The efficacy of this vaccine was investigated in a controlled comparative trial against challenges with two strains of *Mhyo* and a PCV2d field isolate, respectively, next to major competitor products.

## 2. Materials and Methods

The presented trials were approved under the NO/JAO/18-4/2021 registration number ethical committee permission. Two consecutive studies (Study A with *Mhyo* and Study B with PCV2d challenge) were performed. The piglets originated from the same source farm of high health status, meaning that it was free of the major swine pathogens, including Aujeszky’s disease virus, brucellosis, PRRSV, and *M. hyo*. Based on the lack of PCV2-associated diseases (PCVADs) over the last few years, the PCV2 pressure was considered low.

**Figure 1:**
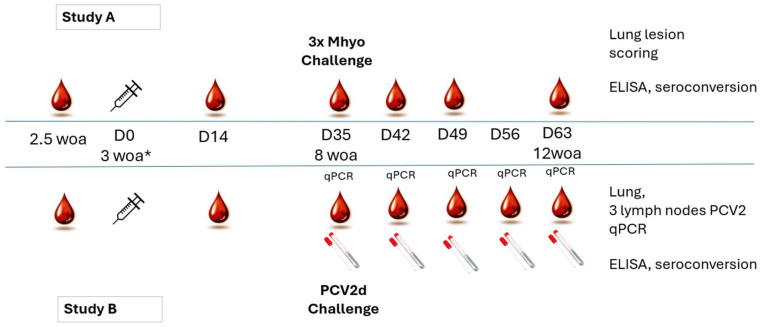
Outline of the two studies. The key activities (bleeding, vaccination, sample collection) and collected sample types (blood, rectal swabs, and organ samples) are indicated (*:woa = weeks of age).

Approximately 18 day-old piglets were transferred from the source farm to the trial facilities, and after randomization for weight, litter, and gender, they were allocated into seven groups with 20 animals in each, as shown in Table 1. At 3 weeks of age, each of them were vaccinated or sham vaccinated according to the manufacturer’s instructions. The scheme of the trials is shown in Figure 1.

### 2.1. Clinical Parameters (Only in Study A)

Animals were under regular veterinary control during the experiment. All individual treatments if needed were recorded. There were two and five mortalities in Studies A and B, respectively, a consequence of an intercurrent disease and not related to the vaccinations and the challenge. Following the challenge, clinical signs related to *Mhyo* were evaluated daily and aggregated weekly until the trial concluded. As the main parameter, coughing was recorded as follows: presence 1, absence 0.

### 2.2. Serology

IDVet ID Screen^®^ *Mycoplasma hyopneumoniae* Competition ELISA kit, BioChek Porcine Circovirus type 2 Antibody test kit ELISA, Ingezim Circovirus IgM/IgG (11.PCV.K2), and Ingezim Circo IgG (11.PCV. K.1) kits were used to measure seroconversion at the indicated sampling timepoints according to manufacturer’s instructions against *Mhyo* and PCV2 antigens.

### 2.3. qPCR (Only in Study B)

PCV2 genome DNA from the sera, rectal swab, organ and oral fluid sampleswas quantified by qPCR as described earlier [13,14,15].

### 2.4. Challenge

At 5 weeks post-vaccination (pv), at 8 weeks of age, all the animals in Study A (except the negative controls) were challenged with *Mhyo* on three consecutive days as follows: 10 mL of challenge material, SZ1V Ghent University strain (courtesy of Prof. Dominiek Maes, Ghent University) was applied intratracheally: 6.8 × 10^7^ Colour Change Unit (CCU)/pig, L1 Ceva-Phylaxia strain: 5 × 10^8^ CCU/pig, and 10^8^ CCU/pig.

The animals in Study B (except the negative controls) were challenged intranasally (3–3 mL per nostril) by Nasal MAD atomizer (MAD Nasal^™^ Intranasal Mucosal Atomization Device (Teleflex, Ireland)) with the PCV2d strain: D3276/5/17 PCV2d (7.8 log10 genome copies/µL, propagated on PK-15 cells) as described earlier [15].

### 2.5. Mhyo

Lung lesion scoring: Extension of EP-compatible lung lesions and presence of fissures and pleurisy were recorded at slaughter according to the European Pharmacopeia 10.0 2448 “Porcine Enzootic Pneumonia Vaccine (Inactivated)” and Hannan et al., 1982 [16].

### 2.6. Statistics

The clinical scores on 49 and 56 postimmunization (dpi) were compared using the χ^2^ test.

Weighted lung lesion scores were compared with a mixed model with multiple comparisons and Sidák correction [17].

The total virus load in sera (viremia), rectal swabs (rectal shedding), and oral fluid samples (shedding through saliva) was computed with AUC using the trapezoid rule, and the groups were compared with a mixed model with multiple comparisons and Sidák correction.

For all statistics performed, the significance level was set to 0.05. Statistical analysis was performed with SAS 9.4.

## 3. Results

### 3.1. Clinical Observations

Study A. The only clinical sign was coughing after *Mhyo* challenge. At 42 and 63 dpi, no clinical signs were observed in any of the groups (Table 2), and consequently only 49 and 56 dpi data were analyzed statistically. At 3 wpc (56 dpi), all the vaccinated groups were found to be significantly prevented from coughing (*p* < 0.001), while almost all of the positive controls showed this clinical sign. At 49 dpi, no statistically significant difference was found.

The challenge in the PCV2 study (Study B) did not induce any clinical alterations.

### 3.2. Serology

#### 3.2.1. *M. Hyopneumoniae* ELISA (Study A and Study B)

No seropositivity was detected before vaccination. Only the same three vaccines (Vaccine A, Vaccine D, and Vaccine F) plus Vaccine B (only applied in Study A) showed seropositivity three weeks post-vaccination. Just before the challenge, only Vaccine A, Vaccine B (Study A), and Vaccine D crossed the positivity limit in both experiments.

**Figure 2:**
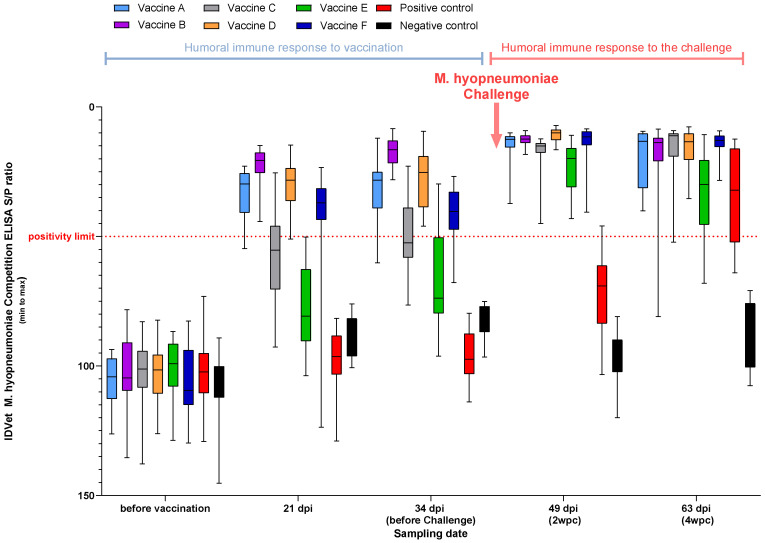
Sample-to-positive (S/P) ratios in the serum of pigs over time using IDVet ID Screen^®^ *M. hyopneumoniae* Competition ELISA (below 50 S/P is considered positive; Study A).

Seroconversion for the remaining vaccinates was demonstrated at 2 wpc, while the positive control was only demonstrated by the end of the experiment (Figure 2).

In Study B, only the same three vaccines (Vaccine B was not part of in this experiment) showed measurable titers over the positivity limit at 21 dpi.

#### 3.2.2. PCV2 ELISA, Study B

PCV2-specific antibodies declined below the detectable level by the time of challenge (34 dpi) in the control group.

**Figure 3:**
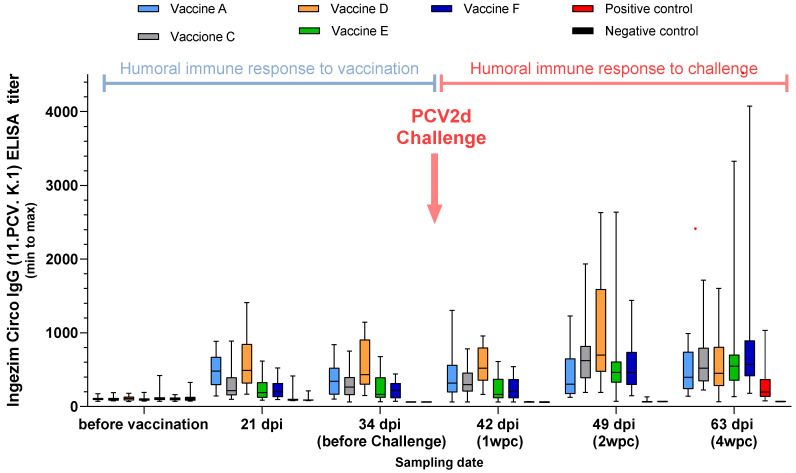
PCV2 serum titers in pigs over time using Ingezim Circo IgG (11.PCV. K.1) ELISA (Study B).

The earliest and most robust seroconversation against PCV2 was observed for the Vaccine A and Vaccine D groups by the Ingezim Circo IgG (11.PCV. K.1) ELISA (Figure 3).

**Figure 4:**
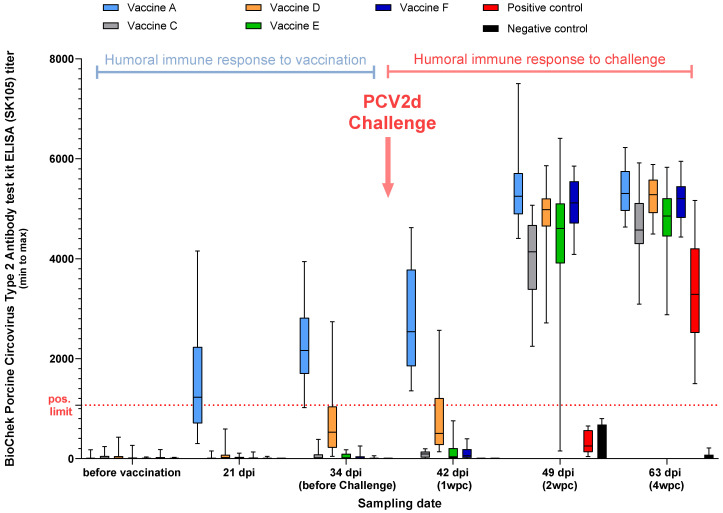
PCV2 serum titers in pigs over time using BioChek Porcine Circovirus Type 2 Antibody test kit ELISA (SK105) ELISA (titer above 1071 is considered positive; Study B).

Comparing the vaccine groups using the BioChek Porcine Circovirus Type 2 Antibody test kit ELISA (SK105), only Vaccine A crossed the positivity limit of the test at 21, 34, and 42 dpi (Figure 4).

#### 3.2.3. IgG/IgM Ratio, Study B

At 1 wpc after challenge, Vaccine A demonstrated 100% anti-PCV2 IgG positivity, the highest among the vaccinates. At 28 days post-vaccination, 95% IgM positivity was seen in the challenged control group, while almost all the vaccinates were negative, showing the fresh infection in the control group.

### 3.3. Mhyo Related Lung Lesions (Study A)

**Figure 5:**
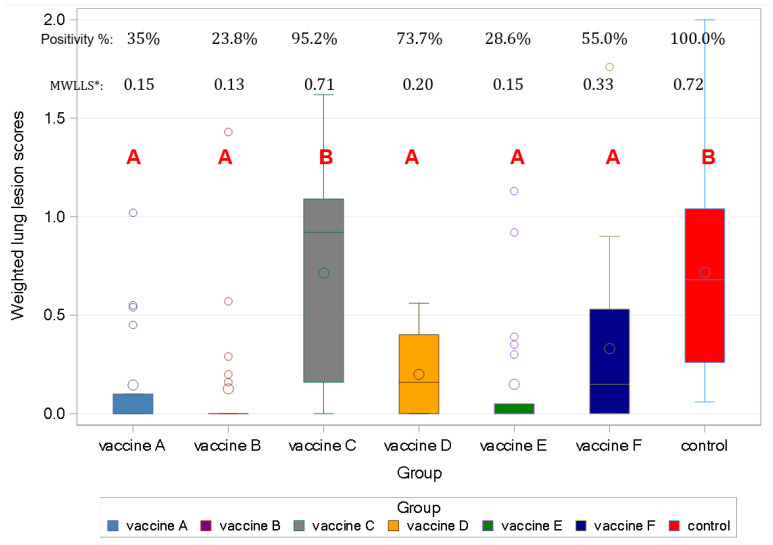
Mean weighted lung lesion scores (MWLLSs) after *Mhyo* challenge, positivity rate, and Box and Whisker’s plot of individual weighted lung lesion scores. (Different capital letters indicate statistically significant differences. *: MWLLS = Mean weighted lung lesion score per each group as described at Section 2.5. *Mhyo* lung lesion scoring.)

All of the pigs in the control group were found to be positive for *M. hyo*-related lung lesions (Figure 5).

All of the vaccines, but Vaccine C, reduced lung lesions significantly, considering weighted lung scores (Figure 5).

### 3.4. PCV2 qPCR—Study B

#### 3.4.1. PCV2 Viraemia

**Figure 6:**
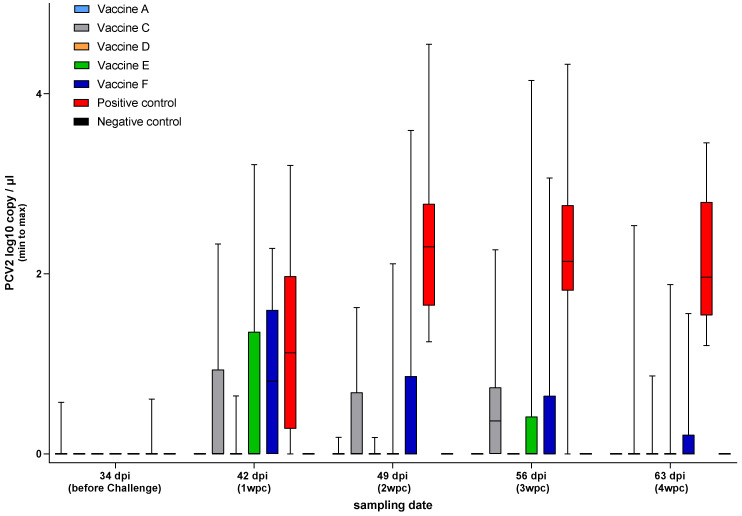
Viral load in the sera after PCV2 challenge (log10 copy/µL).

Serum samples were tested for PCV2 viremia at weekly intervals wpc (Figure 6).

**Figure 7:**
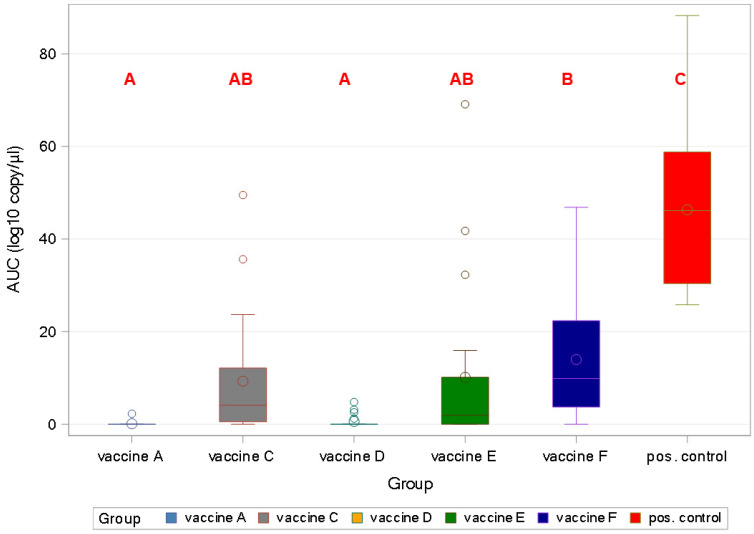
Box and Whisker’s plot of viraemia among groups by area under the curve (AUC) using mixed model (different capital letters indicate statistically significant differences).

All of the vaccines gave significant protection against viral load in sera (*p* < 0.001). Vaccine A and Vaccine D were the only products that protected against viremia completely, as demonstrated by the area under the curve (AUC) calculation (Figure 7).

#### 3.4.2. PCV2 Shedding

**Figure 8:**
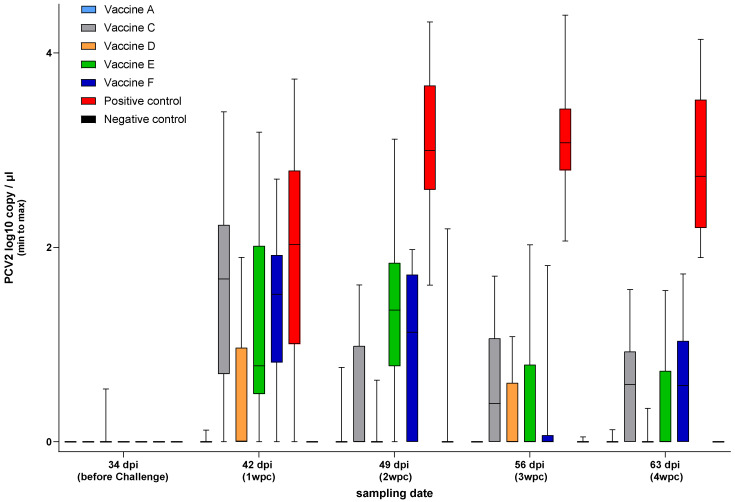
Weekly rectal shedding of PCV2 challenge virus by qPCR (log10 copy/µL).

PCV2 fecal shedding was tested by rectal swabbing and qPCR measurements. Unlike the other vaccines, Vaccine A prevented fecal shedding to levels below the detection limit of the applied methodology (Figure 8).

**Figure 9:**
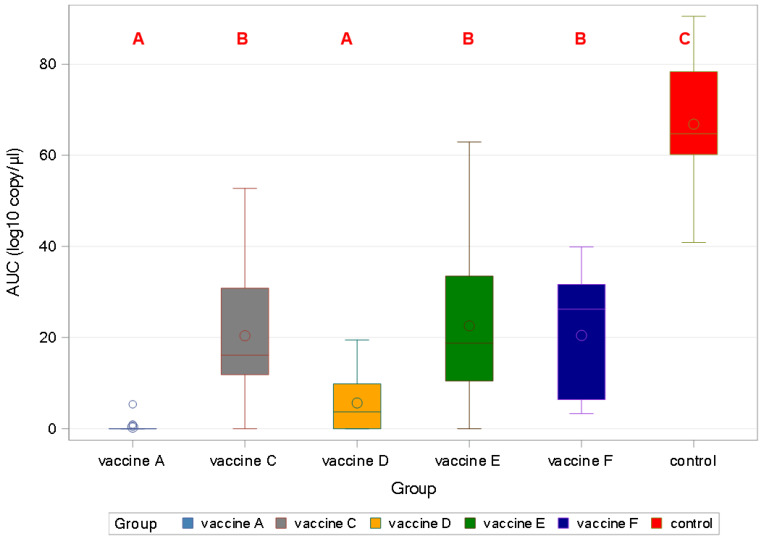
Box and Whisker’s plot of rectal shedding among groups by area under the curve (AUC) using a mixed model (different capital letters indicate statistically significant differences).

All the vaccines gave significant protection against rectal shedding (*p* < 0.001), with Vaccine A and Vaccine D demonstrating better efficiency than the rest (Figure 9).

#### 3.4.3. Oral Fluid qPCR Results

**Figure 10:**
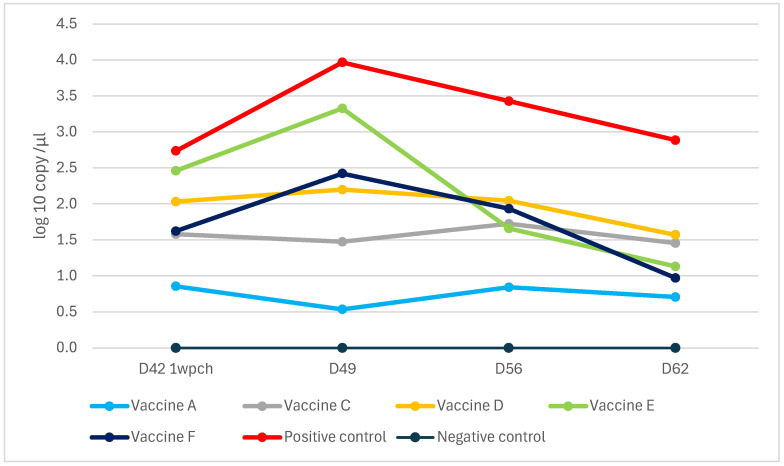
Viral load in oral fluid samples collected weekly after PCV2d challenge (log10 copy/µL) (1 wpc = 1 week post-challenge).

The highest virus level was detected in the positive control at all timepoints and the lowest was detected in the Vaccine A group (Figure 10).

All vaccines decreased the viral load in oral fluid samples significantly (*p* < 0.001).

**Figure 11:**
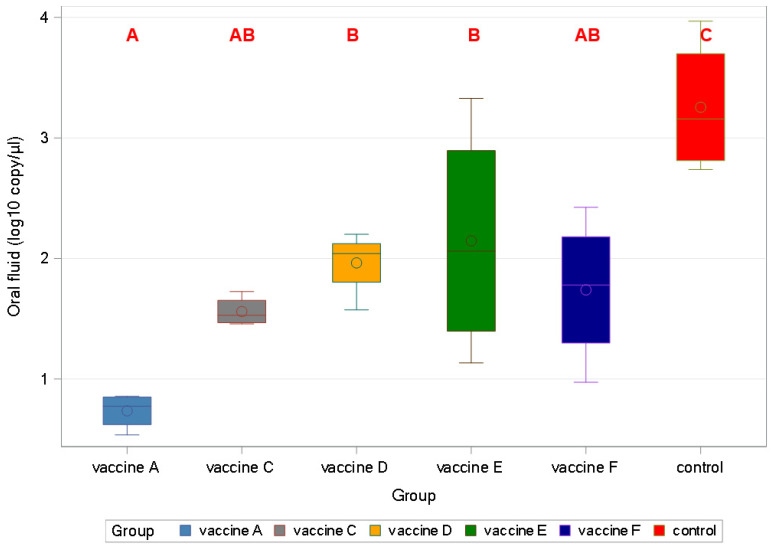
Shedding differences among the groups by area under the curve (AUC) using a mixed model in oral fluid samples (different capital letters indicate statistically significant differences).

Vaccine A proved to be more effective than Vaccine D, followed by Vaccine E (Figure 11).

#### 3.4.4. Viral Load in Organ Samples

**Figure 12:**
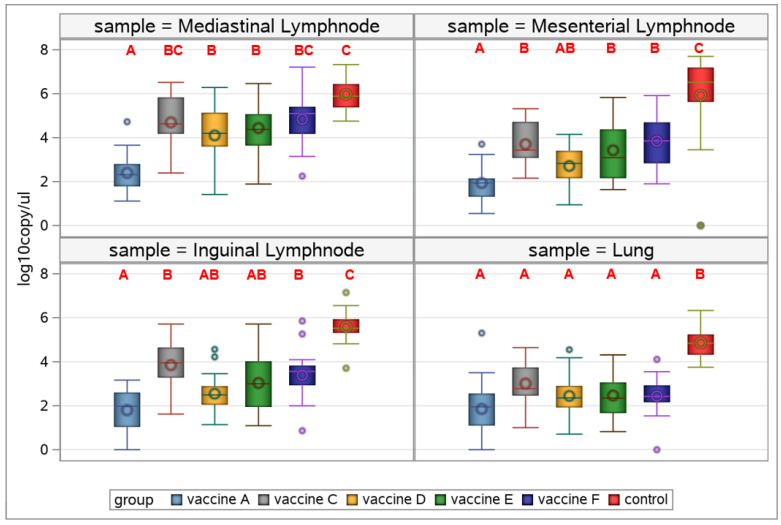
PCV2 log 10 copy number/µL in the organ samples (mediastinal, mesenteric, and inguinal lymph nodes and lungs) collected 4 weeks after challenge (different capital letters indicate statistically significant differences).

Almost all vaccines managed to decrease viral load significantly in all samples collected in contrast/compared with the control group (Figure 12).

In the lymph nodes, Vaccine A was to be the most efficacious to reduce PCV2 load, differing significantly from all (mediastinal lymph nodes), or most (mesenterial lymph nodes), competitor products, while although having a numerically lower group mean value, there was no statistically significant difference between the inguinal lymph node viral loads of Vaccines A, C, and D. Noticeably, lung samples did not show statistically different viral loads among vaccine groups (Figure 12).

## 4. Discussion

Combatting PRDC is one of the major challenges for economic pig production globally. Several pathogens contribute to its conditions, primarily *Mhyo*, PCV2, PRRSV, and swine influenza virus [3]. The prevalence of the pathogens and their specific variants (e.g., virulence, genotype, subtype) influence the manifestation and severity of the disease, and appropriate vaccination provides significant protection against the detrimental impacts of PRDC. Although there are several reports on the evaluation of combined PCV2 and *Mhyo* vaccines, either in controlled or field trials [7,18,19], this is the first report about the efficacy of a ready-to-use combo vaccine based on a PCV2d strain. It represents the most prevalent and clinically relevant PCV2 genotype lately [9], accompanied by an *Mhyo* strain with a history of proven effectiveness both experimentally and in the field against both enzootic pneumonia and PRDC [20,21,22]. The vaccine and its main competitors were tested against the challenge with the following respective pathogens: PCV2d and a combination of two *Mhyo* strains.

Although no direct correlation is clear between vaccine-induced seroconversion against *Mhyo* and vaccine efficacy, a higher proportion of seroconverting animals indicates a good quality of vaccination, reflecting the immune system’s response to the vaccine [19]. On the other hand, for PCV2 there is a more direct association between seroconversion and vaccine efficacy [23,24].

However, it is important to note that while seroconversion is a good indicator of vaccine efficacy, it is not the only factor. The overall immune response, including cellular immunity, determines the outcome of PCV2 infection [3].

Measured three weeks post-vaccination, a few groups demonstrated seroconversion against *Mhyo*, including those two with the same *Mhyo* antigen, either solely or in combination with PCV2d (Figure 2), demonstrating their ability to induce the humoral immune response early. Only these two groups and Vaccine D and F had their mean titers above the positivity threshold five weeks post-vaccination at the time of the challenge. However, the challenge induced a unanimous titer rise in all vaccinated groups. Vaccine E had the lowest mean titer among the vaccinates throughout the trial, close to the non-vaccinated challenged controls, only crossed the positivity limit after the challenge at the end of Study A.

The two ELISAs used to measure PCV2 seroconversion demonstrated big differences concerning standard deviation. Vaccine A and D showed the earliest and most robust seroconversion against PCV2 with Ingesim Circo IgG kit. Nonetheless, measured with BioChek ELISA the Vaccine A group was the earliest to seroconvert, the only one before challenge, and for the rest of the groups only the challenge could raise the antibody titers above the kit’s threshold. These findings indicate the high immunogenicity of Vaccine A.

Seven days post-challenge, IgM/IgG positivity was in alignment with vaccination status, meaning that few vaccinates were IgM-positive while most of them demonstrated IgG positivity. Four weeks post-challenge, IgM positivity ratio demonstrated efficient replication and antigen expression of the challenge virus in the non-vaccinated challenged control group, while all vaccinated groups had 100% IgG positivity at the end of this study.

Significant protection against *Mhyo*-induced lung lesions was demonstrated for all except one vaccinated group according to the scoring (Figure 5).

A reduction in viremia is considered a major parameter for PCV2 vaccine efficacy, since it was demonstrated that high PCV2 viremia may be associated with the development of PCVAD [25].

Concerning viral load, the highest values were measured for the oral fluid samples with a peak at two weeks post-challenge (Figure 10 and Figure 11). The lowest AUC was calculated for the Vaccine A group, indicating the least viral burden to pen-mates and the environment.

In agreement with a previous study utilizing a similar challenge model [14], lower viremia-related PCV2 copy numbers were measured than for rectal shedding, as represented by the control group. The peak was the second- and third-week post-challenge for viremia and shedding, respectively, according to the sampling schedule. The vaccines proved more efficacious in reducing viremia than shedding. Importantly, the new PCV2d combo vaccine practically prevented both, excelling among the vaccines (Figure 6, Figure 7, Figure 8, Figure 9, Figure 10, Figure 11 and Figure 12).

Beyond viremia, we measured the challenge virus load in different regional lymph nodes and lung samples to follow the spread and replication of the virus within the body. The Vaccine A group exhibited the lowest mean viral loads in organs, with the mediastinal lymph nodes showing significantly lower levels than any other group, indicating strong vaccine efficacy (Figure 12), which affects overall health status and growth rates through reducing the immunosuppressive effects of the virus, and associated treatment and management costs [26]. It is of note that lung samples would not allow the scope of differentiation as the lymph nodes.

Although they were beyond the scope of the presented studies, genetic factors play a role in the outcome of either PCV2 and Mhyo infections [27,28], which should also be addressed in future investigations to obtain a better picture of protection/resistance against these pathogens.

Finally, the oral fluid results encourage stakeholders of the swine industry to utilize this methodology more frequently, since this minimally invasive sampling approach presents less or no stress to the animals, requires reduced handling from farm personnel, even if limited, provides environmental enrichment to the pigs, and yet provides valuable health-related data.

## 5. Conclusions

Both studies A and B demonstrated the exceptional efficacy of the new PCV2d *Mhyo* combo vaccine. Its strengths include the following. (i) Significant reduction in Mhyo-induced clinical signs: the vaccine effectively mitigates the clinical symptoms associated with Mhyo infections, leading to healthier pigs with fewer respiratory issues. (ii) Early seroconversion to both vaccine antigens: it indicates the timely reaction of the immune system to the vaccination. (iii) It demonstrated significant reduction in *Mhyo*-induced lung lesions, which results in healthier pigs with fewer respiratory issues and potential secondary infections. (iv) It demonstrated suppression of PCV2 viremia and shedding (both orally and via feces) below the detection limit, which is vital for controlling the spread of the virus within and between herds. (v) Finally, it demonstrated a significant reduction in organ viral load in experimentally challenged animals, demonstrating its ability to control systemic infection and reduce the overall viral burden in the body.

Overall, the findings from these studies indicate that the PCV2d Mhyo combo vaccine is a highly effective tool in combating PRDC and PCVAD. By addressing both PCV2 and Mhyo infections, the vaccine offers comprehensive protection, improving the health and productivity of swine herds.

## Figures and Tables

**Table 1:** Group assignments and details of the vaccines used in Studies A and B.

Group	G1	G2 (Only in Study A)	G3	G4	G5	G6	G7 Challenged Control	G8 Negative Unchallenged Control *
Product name	Cirbloc M Hyo	Hyogen	FLEXcombo	Porcilis PCV M Hyo	CircoMax Myco	Mhyosphere PCV ID		
	Vaccine A	Vaccine B	Vaccine C	Vaccine D	Vaccine E	Vaccine F		
Composition	PCV2d capsid protein + inactivated *M. hyo* strain 2940	Inactivated *M. hyo* strain 2940	PCV-2 recombinant ORF2 protein + Inactivated *M. hyo* J Strain Isolate B-3745	PCV2 ORF2 subunit antigen + Inactivated *M. hyo* J Strain	Inactivated recombinant chimeric PCV1 containing PCV2a and 2b ORF2 subunit antigens, plus inactivated *M. hyo* strain P-5722-3	Inactivated recombinant *M. hyo*^cpPCV2^ strain Nexhyon	PBS	PBS
Dose	2 mL	2 mL	2 mL	2 mL	2 mL	0.2 mL	2 mL	2 mL
Administration route	i.m.	i.m.	i.m.	i.m.	i.m.	i.d.	i.m.	i.m.
Animals	20	20	20	20	20	20	20	10

*: Negative control served validation purposes and was not included in statistical analysis. i.m. = intramuscular; i.d. = intradermal.

**Table 2:** Ratio of animals coughing.

	42 dpi	49 dpi	56 dpi	63 dpi
Vaccine A	0%	5%	15%	0%
Vaccine B	0%	4.8%	9.5%	0%
Vaccine C	0%	14.3%	19%	0%
Vaccine D	0%	10%	15%	0%
Vaccine E	0%	14.3%	14.3%	0%
Vaccine F	0%	10%	15%	0%
Positive control	0%	26.3%	89.5%	0%
Negative control	0%	0%	0%	0%

## Data Availability

Data related to the manuscript are available from the authors upon reasonable request.

## Abbreviations

The following abbreviations are used in this manuscript:

EP	Enzootic pneumonia
PRDC	Porcine respiratory disease complex
PCV2	Porcine circovirus type 2
PCVAD	Porcine circovirus-associated diseases
